# Synthesis, Molecular Docking and Biological Evaluation of Glycyrrhizin Analogs as Anticancer Agents Targeting EGFR

**DOI:** 10.3390/molecules19056368

**Published:** 2014-05-19

**Authors:** Yong-An Yang, Wen-Jian Tang, Xin Zhang, Ji-Wen Yuan, Xin-Hua Liu, Hai-Liang Zhu

**Affiliations:** 1 State Key Laboratory of Pharmaceutical Biotechnology, Nanjing University, Nanjing 210093, China; E-Mails: yangyan73@163.com (Y.-A.Y.); prczhangxin@163.com (X.Z.); 363718480@163.com (J.-W.Y.); xhliuhx@163.com (X.-H.L.); 2 School of Pharmacy, Anhui Medical University, Hefei 230032, China

**Keywords:** glycyrrhizin, anticancer activity, EGFR, 18-epimer, molecular docking

## Abstract

Glycyrrhizin (GA) analogs in the form of 3-glucuronides and 18-epimers were synthesized and their anticancer activities were evaluated. Alkaline isomerization of monoglucuronides is reported. *In vitro* and *in vivo* studies showed that glycyrrhetinic acid monoglucuronides (GAMGs) displayed higher anticancer activities than those of bisglucuronide GA analogs, while anticancer activity of the 18*α*-epimer was superior to that of the 18*β*-epimer. 18*α*-GAMG was firstly nicely bound to epidermal growth factor receptor (EGFR) via six hydrogen bonds and one charge interaction, and the docking calculation proved the correlation between anticancer activities and EGFR inhibitory activities. Highly active 18*α*-GAMG is thus of interest for the further studies as a potential anticancer agent.

## 1. Introduction

The epidermal growth factor receptor (EGFR) is a transmembrane glycoprotein that defines a family of tyrosine kinase receptors (TKRs) including ErbB2/HER2, ErbB3/HER3 and ErbB4/HER4 [1,2]. As a cell surface protein that binds to epidermal growth factor, its binding to a ligand induces receptor dimerization and tyrosine autophosphorylation and leads to cell proliferation, of which altered activity has been implicated in the development and growth of many tumors [3]. EGFR is highly expressed in adult hepatocytes and the EGFR family plays a central hepatoprotective and pro-regenerative role in the liver [4,5]. Mice lacking EGFR or heparin-binding EGF show delayed regeneration after partial hepatectomy (PH), which demonstrates that EGFR is a critical regulator of hepatocyte proliferation during liver regeneration [6,7]. The treatment of hepatocellular carcinoma (HCC) cells with EGFR-specific tyrosine kinase inhibitors or neutralizing antibodies induces cell cycle arrest and apoptosis and increases chemosensitivity [8,9]. Hence, EGFR has long been an attractive candidate as anticancer drug target. Over the past 30 years, much effort has been directed at developing anticancer agents that can interfere with EGFR activity, such as, monoclonal antibodies and small-molecule inhibitors.

Natural products play a major role in drug discovery, and nearly half of the new drugs introduced into the market over the past two decades are natural products or their derivatives [10]. The roots and rhizomes of licorice (*Glycyrrhiza*) species have long been used worldwide as a herbal medicine and natural sweetener. Glycyrrhizin (Glycyrrhizic acid, GA, 18*β*-GA), the major bioactive compound in licorice, is developed as a drug with multi-pharmacological effects such as anti-inflammation, antivirus, anti-tumor, and immuno-modulating properties, among others [11,12,13]. GA has been used in Japan for more than 60 years as a treatment for chronic hepatitis C, thus long-term administration was effective in preventing hepatic cirrhosis and HCC [14,15,16]. GA exhibits hepatoprotective activity by decreasing serum liver enzyme levels and improving tissue pathology in hepatitis patients, while *in vitro* studies showed that its anticancer activity is achieved by inhibiting abnormal cell proliferation, tumor formation and growth [17,18,19,20].

**Figure 1:**
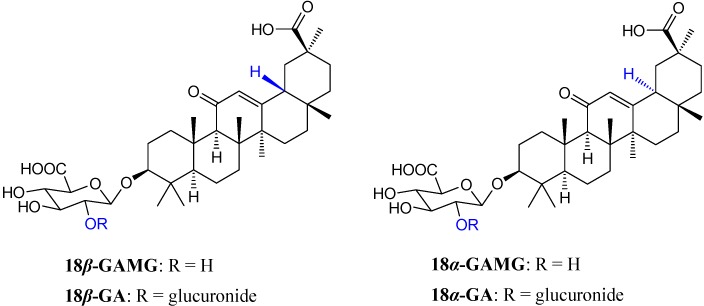
Glycyrrhizin analogs with 3-glucuronides and 18-epimers.

GA is a conjugate of an 18*β*-H-oleanane-type aglycone and two glucuronic acids at the C-3 position, and it could be transformed into 18*β*-glycyrrhetinic acid monoglucuronide (18*β*-GAMG) by removing one terminal glucuronic acid [21,22]. Compared to GA, 18*β*-GAMG showed similar (or stronger) pharmacological activities, such as antitumor, antivirus, anti-allergic, and anti-inflammatory activities [23,24]. 18*α*-Glycyrrhizin (18*α*-GA), a D/E-*trans*-epimer, was prepared by alkaline isomerization of 18*β*-GA [25]. 18*α*-GA also showed similar anti-inflammatory and anticancer activity [26,27]. Researches showed that GA analogs are primary hepatocyte mitogens that bind to EGFRs and subsequently stimulate the receptor tyrosine kinase mitogenactivated protein kinase pathway to induce hepatocyte DNA synthesis and proliferation [28]. Herein, we prepared glycyrrhizin analogs (Figure 1) and further evaluated their anticancer activities *in vitro* and *in vivo*. 

Based on the EGFR complex structure (PDB Code 1M17) [29], computer-generated docking molecular models of GA analogs were analyzed using the Discovery Studio 3.5, and we also initiated an effort to leverage molecular modeling in combination with available data to study the effect of structure of glycyrrhizin on anticancer activity.

## 2. Results and Discussion

GA (18*β*-GA) was provided by Jiangsu Tian Sheng Pharmaceutical Co. Ltd., Jiangsu, China. *Aspergillus* sp Ts-1, a kind of *β*-glucuronidase, selectively hydrolyzed the terminal–glucuronyl linkage of 18*β*-GA to produce 18*β*-GAMG in 54% yield. 18*α*-GA and 18*α*-GAMG were respectively synthesized in yields of 63% and 71% after recrystallization from 18*β*-GA and 18*β*-GAMG by alkaline isomerization. The isomerization reaction was monitored by ^13^C-NMR spectroscopy, and the structure of 18*α*-GAMG was elucidated by comparison with ^1^H-NMR and ^13^C-NMR data of 18*β*-GAMG [30] (see Supplementary Information).

Anti-proliferative activities of glycyrrhizin analogs and erlotinib against the HepG2 (hepatocellular carcinoma), HeLa (cervix of uterus adenocarcinoma) and A549 (lung carcinoma), were evaluated by CCK8 dye assays. The results, summarized in Table 1, revealed that four glycyrrhizin analogs exhibited significant antitumor activities. Two monoglucuronide compounds, 18*α*-GAMG and 18*β*-GAMG, exhibited more significant antitumor activities than those with bisglucuronide GAs, while the antitumor activity of the 18*α*-epimer was superior to that of 18*β*-epimer for identical cell lines. Among the four analogs, 18*α*-GAMG displayed the most potent activity, with IC_50_ values of 6.67, 7.43 and 15.76 μM against HepG2, Hela and A549, respectively.

**Table 1:** *In vitro* anticancer activities (IC_50_, μM) of title compounds against human tumor cell lines.

Compd.	IC_50_, (μM) ^a^		
	HepG2 ^b^	HeLa ^b^	A549 ^b^
18*α*-GAMG	6.67	7.43	15.76
18*β*-GAMG	33.60	8.39	21.55
18*α*-GA	54.24	15.13	41.57
18*β*-GA	63.59	18.93	51.92
Erlotinib	0.12	0.20	0.13

**^a^** Antiproliferation activity was measured using the CCK-8 assay. Values are the average of three independent experiments run in triplicate. Variation was generally 5%–10%; **^b^** Cancer cells kindly supplied by State Key Laboratory of Pharmaceutical Biotechnology, Nanjing University.

To generate data concerning the broad spectrum potential of these compounds in Table 2, the IC_50_ values of synthesized compounds against EGFR enzymes are summarized in Table 2. Reference data for erlotinib had also been included for comparison with the compounds reported in this study. For the majority of the compounds, we found that compound 18*α*-GAMG, with an IC_50_ of 0.028 μM, was a better inhibitor than the positive control erlotinib with an IC_50_ of 0.030 μM, suggesting that, at least in part, inhibition of proliferation of the these lines may be the result of EGFR inhibition.

**Table 2:** Data of the *in vitro* EGFR (IC_50_, μM) enzyme inhibition assay of the synthesized compounds.

Compd.	EGFR (IC_50_, μM) ^a^	Compd.	EGFR (IC_50_, μM) ^a^
18*α*-GAMG	0.028	18*β*-GA	0.092
18*β*-GAMG	0.069	Erlotinib	0.030
18*α*-GA	0.081		

**^a^** Minimum cytotoxic concentration required to cause a microscopically detectable alteration of normal cell morphology.

In order to gain more understanding of the structure–activity relationships observed at the EGFR, molecular docking of the most potent inhibitor 18*α*-GAMG and EGFR was performed on the binding model based on the EGFR complex structure (PDB Code 1M17) using the Discovery Studio 3.5 software [29]. The docking calculation of the analogs was depicted in Table 3, and as shown in Table 3, all analogs had nice binding affinity to EGFR and four analogs' -EDOCKER_ INTERACTION_ ENERGY had the same trend as the anti-proliferative activities, which further proved the correlation between the anti-proliferative activities and EGFR inhibitory activities of the analogs.

**Table 3:** -EDOCKER_INTERACTION_ENERGY of title compounds and 1M17.

Compd.	-EDOCKER_INTERACTION_ENERG ΔG (kcal/mol)
18*α*-GAMG	72.0274
18*β*-GAMG	66.9106
18*α*-GA	58.7009
18*β*-GA	58.6731
Erlotinib	44.3732

**Figure 2:**
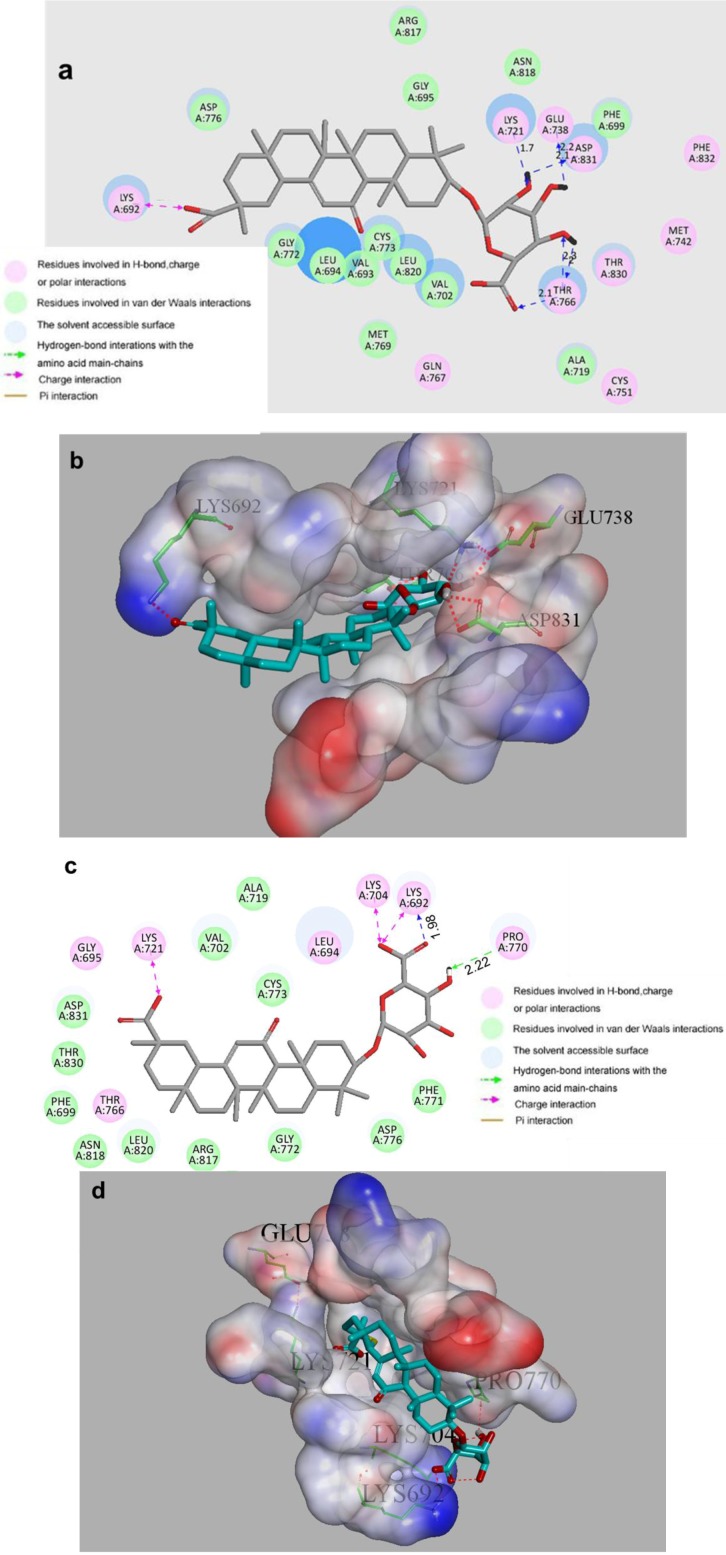
(**a**) 2D molecular docking modeling of 18*α*-GAMG with 1M17. (**b**) 3D model of the interaction between 18*α*-GAMG and 1M17 site. (**c**) 2D molecular docking modeling of 18*β*-GAMG with 1M17. (**d**) 3D model of the interaction between 18*β*-GAMG and 1M17 site.

In the result of molecular docking, 18*α*-GAMG showed maximum -EDOCKER_ INTERACTION_ENERGY, which suggested it was mostly easy to bind to EGFR. The 2D and 3D binding models of 18*α*-GAMG with EGFR are depicted in Figure 2. The amino acid residues which had interactions with EGFR as well as bond lengths were labeled. In the binding models, 18*α*-GAMG was nicely bound to EGFR via six hydrogen bonds with ASP831 (angle = 120.49°, distance = 2.14 Å), GLU738 (angle = 142.28°, distance = 2.2 Å), THR766 (three bonds: angle = 140.28°, distance = 2.1 Å; angle = 117.6°, distance = 2.3 Å; angle = 152.94°, distance = 2.0 Å) and LYS721 (angle = 179.02°, distance = 1.7 Å). In addition, compound 18*α*-GAMG was also nicely bound to EGFR via one charge interaction. The end group of LYS692 formed one charge interaction with a carboxyl which strengthened the binding affinity, leading to the increased anticancer activities of 18*α*-GAMG. Besides, the hydrogens of LYS692, LYS692 and PRO770 formed three hydrogen bonds interaction with the amino group nitrogen atom of 18*β*-GAMG (angle H-N_LYS692_ O35 = 151.7°, distance = 1.98 Å, angle H_ LYS692_ O36 = 123.6°, distance = 2.47 Å, angle H96 O_ PRO770_ = 113.4°, distance = 2.22 Å). Furthermore, compound 18*β*-GAMG was also nicely bound to EGFR via three charge interactions. The end group of LYS692, LYS704 and LYS721 respectively formed three charge interactions with two carboxyls. These molecular docking results, along with the biological assay data, suggest that compound 18*α*-GAMGpossesses higher anticancer activity than 18*β*-GAMG, which will help us carry out structure optimization based on computer-aided design.

Recently, 18*β*-GA has been recognized as a hepatoprotective high-mobility group protein 1 (HMGB1) inhibitor, which binds directly to both HMG boxes in HMGB1 and attenuates HMGB1-induced hepatocyte apoptosis, thus leading to induce hepatocyte DNA synthesis and proliferation [31,32]. As it is, 18*β*-GA induced hepatocyte proliferation, while we got the opposite results in cancer cells. The binding moiety of 18*α*-GAMG with EGFR was mainly the glucuronide unit, but GA could inhibit HMGB1 by binding of its triterpene ring directly to the two HMG boxes. These results showed that the protein targets and molecular pathways affected by GA may be complicated and heterogeneous.

**Figure 3:**
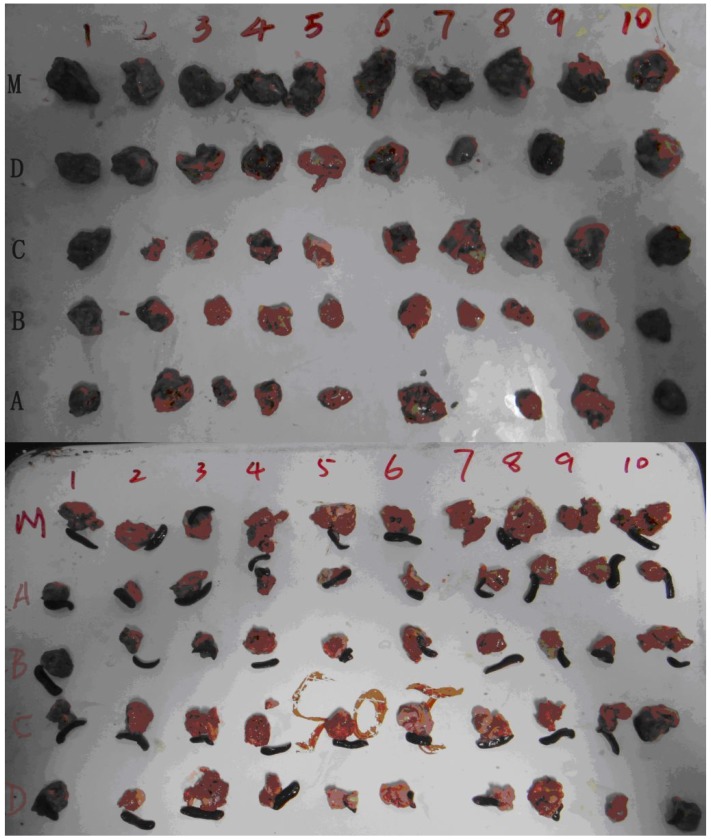
Solid tumors from S180 (above) and HepG2 (below) tumor-bearing mice. A: 18*α*-GAMG; B: 18*β*-GAMG; C: 18*α*-GA; D: 18*β*-GA; M: Normal saline.

To further verify the inhibitory effect of glycyrrhizin analogs on the growth of tumor cells *in vivo*, sarcoma cells S180, hepatoma cells HepG2 and Ehrlich ascites cells EAC were selected to evaluate *in vivo* antitumor effects. The inhibitory effects of glycyrrhizin analogs on the growth of the transplanted S180 or HepG2 carcinoma are presented in Table 4 and Figure 3. The results revealed that glycyrrhizin analogs significantly decreased the tumor weights of S180 and HepG2 tumor-bearing mice. The inhibitory rates of GAMGs were higher than those of GAs, while the inhibitory rate of the 18*α*-epimer was higher than that of corresponding 18*β*-epimer. The most potent activity was showed by 18*α*-GAMG with inhibitory rates 39.8% and 49.7% for S180 and HepG2 tumor-bearing mice at the dosage of 60 mg/kg/day, respectively.

EAC tumor-bearing mice were observed for mean survival time. The effect of glycyrrhizin analogs on percentage increases in life span was calculated on the basis of mortality of the experimental mice. Survival response of untreated EAC-bearing mice died within 16.4 days (Table 5). A similar phenomenon was observed: mice administered monoglucuronide and 18*α*-epimer displayed longer survival times. The 18*α*-GAMG group was observed to enhance the survival rate to 45.4%.

Based on *in vitro* and *in vivo* experiments, followed by molecular docking, we here demonstrated that the protein target Epidermal Growth Factor Receptor (EGFR) was also sensitive to four glycyrrhizin analogs in three types of carcinoma cells, indicative of their potential anticancer activity as the EGFR inhibitors. The result was significant and intriguing, but further studies needs to be provided to systematically elucidate the direct correlation between the glycyrrhizin analogs and the EGFR target, which would reveal the new mechanism of glycyrrhizin action.

**Table 4:** Antitumor effects of glycyrrhizin analogs against tumor growth on the S180 and HepG2 xenograft mice. **^a^**

Models	Groups	Animal number (End, n)	Body weight (g)		Tumor weight (g)	Inhibition rate (%)
			Beginning	End		
	Control	10	19.80 ± 1.32	23.97 ± 2.23	1.91 ± 0.29	
	18*α*-GAMG	9	19.40 ± 1.07	25.25 ± 1.80	1.15 ± 0.50 **	39.8
S180	18*β*-GAMG	10	19.80 ± 1.39	25.02 ± 2.58	1.25 ± 0.19 **	34.6
	18*α*-GA	10	20.00 ± 1.49	25.20 ± 1.11	1.29 ± 0.47 **	32.5
	18*β*-GA	9	19.70 ± 1.25	24.48 ± 2.37	1.33 ± 0.67 **	30.4
	Control	10	19.4 ± 1.35	25.05 ± 1.89	1.95 ± 0.22	
	18*α*-GAMG	10	19.6 ± 1.51	27.02 ± 2.10	0.98 ± 0.43 **	49.7
HepG2	18*β*-GAMG	10	19.00 ± 0.94	27.8 ± 1.57	1.20 ± 0.35 **	38.4
	18*α*-GA	10	20.1 ± 1.45	26.08 ± 1.26	1.22 ± 0.46 **	37.4
	18*β*-GA	10	19.4 ± 1.08	26.94 ± 2.05	1.26 ± 0.65 **	35.4

**^a^** Mice were inoculated with S180 or HepG2 subcutaneously into the right front armpit and randomly divided into five test groups. The mice were daily treated by 18*α*-GAMG, 18*β*-GAMG, 18*α*-GA, 18*β*-GA (60 mg/kg/day), or normal saline (NS, 10 mL/kg) by oral gavage for ten consecutive days. Data were analyzed using SPSS11.0. Significant difference between each treatment and the control are shown as *P* < 0.05 (*) and *P* < 0.01 (**).

**Table 5:** Effects of glycyrrhizin analogs against the survival of EAC-bearing mice.

Groups	Animal number (n)	Body weight (g)	Survival time ^a^ (d)	Survival rate (%)
Control	10	19.0 ± 1.25	16.40 ± 2.07	
18*α*-GAMG	10	19.5 ± 1.27	23.85 ± 5.41*	45.4
18*β*-GAMG	10	19.0 ± 0.82	21.05 ± 4.65*	28.4
18*α*-GA	10	19.4 ± 0.97	19.75 ± 3.08*	20.4
18*β*-GA	10	19.4 ± 1.27	19.40 ± 3.77*	18.3

**^a^** Time denoted by number of days. *P* < 0.05 (*)

## 3. Experimental Section

### 3.1. Synthesis of Glycyrrhizin Analogs

#### 3.1.1. General Methods

*Aspergillus* sp. Ts-1 was isolated from soil collected in Kashi of the Xinjiang Uygur Autonomous Region (China) and selectively hydrolyzed the terminal–glucuronyl linkage of 18*β*-GA to yield glycyrrhetic acid 3-O-mono-*β*-D-glucuronide (18*β*-GAMG). Its subculture and 18*β*-GA were provided by Jiangsu Tian Sheng Pharmaceutical Co. Ltd. (Nanjing, China). All materials were obtained from commercial suppliers, were of analytical reagent grade and used without further purification. Melting points were uncorrected. Silica gel (200–300 mesh, Huanghai, Qingdao, China). TLC: pre-coated silica gel *F_254_* plates. Optical rotations: polar 3002 polarimeter. NMR spectra: Bruker *AV* NMR spectrometer (^1^H: 500 or 300 and ^13^C: 125 or 75 MHz), the residual solvent peaks used as an internal standard, *J* in Hz. TOF- HR MS: Agilent 1260-6221 TOF LC/MS. 

#### 3.1.2. Preparation of 18*β*-GAMG from 18*β*-GA via Biotransformation

18*β*-GA: white powder. Mp 234−236 °C; 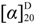 = +52 (*c* = 1.0, MeOH); ^13^C-NMR (75 MHz, DMSO-*d*_6_): Table S1. TOF-HRMS: *m/z* [M + Na]^+^ calcd for C_42_H_62_NaO_16_: 845.3930; found: 845.3935.

*Aspergillus* sp. Ts-1 on glucose yeast agar slant was inoculated into a 250 mL Erlenmeyer flask containing 100 mL of seed medium consisting of 1.0 g glucose, 0.2 g yeast, 1.0 g agar, 0.1 g KH_2_PO_4_ and 0.025g MgSO_4_ in distilled water (pH 7.0). The culture media were sterilized at 121 °C for 20 min and the fermentation was carried out at 30 °C on a rotary shaker at 200 rpm. After 24 h of inoculation, 30 mL sterilized medium was inoculated into a 1,000 mL Erlenmeyer flask containing 300 mL pre-culture sample consisting of 15 g GA, 0.30 g KH_2_PO_4_, 3.0 g urea and 0.24 g MgSO_4_ in distilled water and the pH value was adjusted to 6.0. The culture media were sterilized at 121 °C for 20 min and the fermentation was carried out at 30 °C on a rotary shaker at 250 rpm.

After 72 h of inoculation, the culture solution was filtered and the filtrate was extracted with ethyl acetate. The extract was concentrated under the reduced pressure. The residue (14.5 g) was applied to a silica gel column (800 g, 5.0 × 100 cm) and eluted with CHCl_3_–MeOH in a gradient manner from 100:1 to 1:1. By TLC analysis, fractions I–IX was obtained. Fractions VI–VIII was concentrated *in vacuo* and recrystallization from aqueous MeOH to give 18*β*-GAMG (6.35 g, 54% yield) as a white crystalline powder. Mp 237−239 °C; 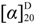 = +91 (*c* = 1.0, MeOH); ^1^H-NMR (500 MHz, DMSO-*d*_6_) *δ* (ppm): 0.76 (s, 3H, 24-CH_3_), 0.77 (s, 3H, 28-CH_3_), 0.99 (s, 3H, 23-CH_3_), 1.06 (s, 2 × 3H, 25-CH_3_, 26-CH_3_), 1.10 (s, 3H, 29-CH_3_), 1.34 (s, 3H, 27-CH_3_), 2.34 (s, 1H, 9-H), 3.01 (m, 1H, 4'-H), 3.08 (dd, 1H, *J*_1_= 4.8 Hz, *J*_2_= 11.2 Hz, 3-H), 3.15 (t, 1H, *J*= 9.0 Hz, 3'-H), 3.30 (m, 1H, overlapped, 2'-H), 3.58 (d, 1H, *J*= 9.7 Hz, 5'-H), 4.25 (d, 1H, *J*= 7.8 Hz, 1'-H), 5.40 (s, 1H, 12-H); ^13^C-NMR (125 MHz, DMSO-*d*_6_): Table S1. TOF-HRMS: *m/z* [M + Na]^+^ calcd for C_36_H_54_NaO_10_: 669.3609; found: 669.3608. 

#### 3.1.3. General Procedure of Alkaline Isomerization of the 18*β*-isomer to the 18*α*-isomer

A solution of 18*β*-isomer (6.0 mmol) in 5.0 M NaOH solution (100 mL) was heated and stirred for 12 h at 90 °C. After the reaction mixture was cooled to <5 °C, the pH was adjusted to 2.5 with concentrated HCl. After 12 h, the mixture was filtrated, washed with water, dried. The product (18*α*-isomer) was obtained by crystallization from ethanol/EtOAc. (Scheme S1 and Figure S1). 18*α*-GA: According to the above procedure, diammonium 18*α*-GA (3.17 g, 63% yield) was obtained from 18*β*-GA (5.00 g) as a white crystalline powder. Mp 211−216 °C; 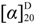 = +20 (*c* = 1.0, MeOH); ^1^H-NMR (300 MHz, DMSO-*d*_6_) *δ* (ppm): 0.65 (s, 3H, 28-CH_3_), 0.73 (s, 3H, 24-CH_3_), 0.95 (s, 3H, 23-CH_3_), 1.04 (s, 3H, 26-CH_3_), 1.10 (s, 3H, 25-CH_3_), 1.16 (s, 3H, 29-CH_3_), 1.33 (s, 3H, 27-CH_3_), 4.31 (d, 1H, *J*= 7.3 Hz, 1'-H), 4.49 (d, 1H, *J*= 7.6 Hz, 1''-H), 5.33 (s, 1H, 12-H); ^13^C-NMR (75 MHz, DMSO-*d*_6_): Table S1. TOF-HRMS: *m/z* [M + Na]^+^ calcd for C_42_H_62_NaO_16_: 845.3930; found: 845.3938.

18*α*-GAMG: 18*α*-GAMG (2.83 g, 71% yield) was obtained from 18*β*-GAMG (4.00 g) as a white crystalline powder. Mp 229−231 °C; 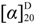 = +24 (*c* = 1.0, MeOH); ^1^H-NMR (300 MHz, DMSO-*d*_6_) *δ* (ppm): 0.65 (s, 3H, 28-CH_3_), 0.77 (s, 3H, 24-CH_3_), 0.92 (s, 3H, 23-CH_3_), 0.98 (s, 3H, 25-CH_3_), 1.04 (s, 3H, 26-CH_3_), 1.16 (s, 3H, 29-CH_3_), 1.33 (s, 3H, 27-CH_3_), 2.27 (overlapped, 9-H), 3.01 (t, 1H,*J*= 8.4 Hz, 4'-H), 3.07 (dd, 1H, *J*_1_= 6.5 Hz, *J*_2_= 9.7 Hz, 3-H), 3.15 (t, 1H, *J*= 9.0 Hz, 3'-H), 3.30 (t, 1H, *J*= 9.8 Hz, 2'-H), 3.58 (d, 1H, *J*= 9.7 Hz, 5'-H), 4.24 (d, 1H, *J*= 7.8 Hz, 1'-H), 5.33 (s, 1H, 12-H); ^13^C-NMR (75 MHz, DMSO-*d*_6_): Table S1. TOF-HRMS: *m/z* [M + Na]^+^ calcd for C_36_H_54_NaO_10_: 669.3609; found: 669.3600.

### 3.2. Biological Assay of in Vitro Anticancer Activities

CCK8 is much more convenient and helpful than MTT for analyzing cell proliferation, because it can be reduced to soluble formazan by dehydrogenase in mitochondria and has little toxicity to cells. Cell proliferation was determined using CCK8 dye (BeyotimeInst Biotech, Shanghai, China) according to manufacturer’s instructions. Briefly, 1–5 × 10^3^ cells per well were seeded in a 96-well plate, grown at 37 °C for 12 h, Subsequently, cells were treated with compounds at increasing concentrations in the presence of 10% FBS for 24 or 48 h. After 10 µL CCK8 dye was added to each well, cells were incubated at 37 °C for 1–2 h and Plates were read in a Victor-V multilabel counter (Perkin-Elmer, Waltham, MA, USA) using the default europium detection protocol. Percent inhibition or IC_50_ values of compounds were calculated by comparison with DMSO-treated control wells. 

### 3.3. General Procedure for Preparation, Purification of EGFR, and Inhibitory Assay

A 1.6 kb cDNA encoded for the EGFR cytoplasmic domain (EGFR-CD, amino acids 645–1186) were cloned into baculoviral expression vectors pBlueBacHis2B and pFASTBacHTc (Huakang Company, Changsha, China), separately. A sequence that encodes (His)_6_ was located at the 5′ upstream to the EGFR sequences. Sf-9 cells were infected for 3 days for protein expression. Sf-9 cell pellets were solubilized at 0 °C in a buffer at pH 7.4 containing 50 mM HEPES, 10 mM NaCl, 1% Triton, 10 µM ammonium molybdate, 100 µM sodium vanadate, 10 µg/mL aprotinin, 10 µg/mL leupeptin, 10 µg/mL pepstatin, and 16 µg/mL benzamidine HCl for 20 min followed by 20 min centrifugation. Crude extract supernatant was passed through an equilibrated Ni-NTA superflow packed column and washed with 10 mM and then 100 mM imidazole to remove nonspecifically bound material. Histidine tagged proteins were eluted with 250 and 500 mM imidazole and dialyzed against 50 mM NaCl, 20 mM HEPES, 10% glycerol, and 1 µg/mL each of aprotinin, leupeptin and pepstatin for 2 h. The entire purification procedure was performed at 4 °C or on ice [29].

EGFR kinase assays were set up to assess the level of autophosphorylation based on DELFIA/Time-Resolved Fluorometry. All compounds were dissolved in 100% DMSO and diluted to the appropriate concentrations with 25 mM HEPES at pH 7.4. In each well, 10 µL compound was incubated with 10 µL (5 ng for EGFR) recombinant enzyme (1:80 dilution in 100 mM HEPES) for 10 min at room temperature. Then, 10 µL of 5 mM buffer (containing 20 mM HEPES, 2 mM MnCl_2_, 100 µM Na_3_VO_4_ and 1 mM DTT) and 20 µL of 0.1 mM ATP–50 mM MgCl_2_ were added for 1 h. Positive and negative controls were included in each plate by incubation of enzyme with or without ATP–MgCl_2_. At the end of incubation, liquid was aspirated, and plates were washed three times with wash buffer. A 75 µL (400 ng) sample of europium labeled anti-phosphotyrosine antibody was added to each well for another 1 h of incubation. After washing, enhancement solution was added and the signal was detected by Victor (Wallac Inc., Gaithersburg, MD, USA) with excitation at 340 nm and emission at 615 nm. The percentage of auto-phosphorylation inhibition by the compounds was calculated using the following formula: 100% − [(negative control)/(positive control − negative control)]. The IC_50 _was obtained from curves of percentage inhibition with eight concentrations of compound. As the contaminants in the enzyme preparation are fairly low, the majority of the signal detected by the anti-phosphotyrosine antibody is from EGFR.

### 3.4. Evaluation of the in Vivo Antitumor Activities

#### 3.4.1. Animals and Cell Lines

Kunming mice (SPF, male or female, 20 ± 2 g) were purchased from the experimental animal center of China Pharmaceutical University. Animals were housed in a temperature (22 ± 2 °C) and relatively humidity (50%)-controlled room on a 12 h light/dark cycle, given free access to food and water, and acclimatized for at least one week prior to use. All the animal experiments were performed in accordance with the Regulations of the Experimental Animal Administration issued by the State Committee of Science and Technology of China.

Cell lines used for evaluation of the *in vivo* antitumor activity in this study included three tumor cell lines, namely S180 (sarcoma tumer cell line), HepG2 (liver carcinoma cell line), EAC (Ehrlich ascites carcinoma cell line). All of cell lines were purchased by the Shanghai Institutes for Biological Sciences, Chinese Academy of Sciences, and the cells were cultured in RPMI-1640 medium, which was supplemented with 10% heat-inactivated fetal bovine serum, 100 U/mL penicillin and 100 U/mL streptomycin and cultured in an atmosphere of 5% CO_2_ at 37 °C. Cells were collected for the experiments in the logarithmic growth phase. 

To establish the tumor-bearing mouse model, the cell lines were harvested and inoculated subcutaneously into the right armpit region of the mice. On the 7th day, the tumor ascrites were obtained and washed with sterile PBS. Under sterile condition, the tumor ascrites were diluted with sterile nomal saline to 1 × 10^10^ /L cell suspension. Tumor ascites were maintained *in vivo* in mice by transplantation of 0.2 mL of ascites (2 × 10^6^ cells) from the infected mice to the non-infected mice.

#### 3.4.2. *In Vivo* Tumor Xenograft Model

Each Kunming mouse (male or female, weight 20 ± 2 g) were inoculated with seven-day-old ascrite (0.2 mL, 2 × 10^6^ cells) subcutaneously into the right front armpit. 24 h after implantation of tumor cells, the mice were randomly divided into five test groups with 10 mice per group. Each mouse was weighed immediately after inoculation. The mice were treated by oral gavage with test samples (60 mg/kg/day) or normal saline (NS, 10 mL/kg) for ten days once daily. On day 11, the mice were sacrificed via cervical dislocation, and the mouse and tumor were excised and weighed for evaluating the tumor growth inhibition. The tumor inhibitory rate was calculated by the following formula:

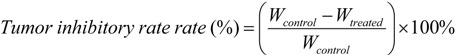
(1)
where W_control_ and W_treated_ were the average tumor weights of the control and treated mice, respectively. EAC tumor-bearing mice were observed for mean survival time. The effect of glycyrrhizin analogs on percentage increases in life span was calculated on the basis of mortality of the experimental mice:


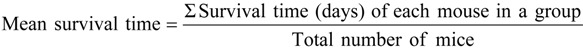
(2)


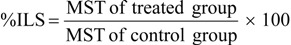
(3)

## 4. Conclusions

In summary, we synthesized glycyrrhizin analogs by glucuronidase biotransformation and alkaline isomerization, and evaluated their biological activities *in vitro* and *in vivo*. Anticancer activities of monoglucuronide GAMGs were higher than those of bisglucuronide GAs, while 1the 8*α*-epimer showed better activity than the 18*β*-epimer. Among them, 18*α*-GAMG displayed the most potent *in vitro* activity, with IC_50_ values of 6.67, 7.43 and 15.76 μM against HepG2, Hela and A549, and *in vivo* activity with inhibitory rates 39.8% and 49.7% for S180 and HepG2 tumor-bearing mice, respectively, and it significantly enhanced the survival rate of EAC tumor-bearing mice to 45.4%. The docking calculations showed that four analogs had better binding affinity to EGFR than the reference compound erlotinib and their binding energy had the same trend as anticancer activities, which further proved the correlation between anticancer activities and EGFR inhibitory activities of these compounds. In the binding model, high active compound 18*α*-GAMG was nicely bound to EGFR via six hydrogen bonds with ASP831, GLU738, THR766 (three bonds), LYS721 and one charge interaction, leading to the increased anticancer activities of 18*α*-GAMG. Therefore, 18*α*-GAMG is of interest for further studies as a potential anticancer agent. Further structural optimization of 18*α*-GAMG is ongoing using a variety of rational design strategies [33].

## Supplementary Materials

Supplementary materials can be accessed at: http://www.mdpi.com/1420-3049/19/5/6368/s1.
