# Supplementary material for: Synthesis, Molecular Docking and Biological Evaluation of Glycyrrhizin Analogs as Anticancer Agents Targeting EGFR
Source: Molecules. 2014 May 19;19(5):6368–81. doi: 10.3390/molecules19056368 (PMC6271220; doi:10.3390/molecules19056368)
Supplement: Supplementary file 1 [file molecules-19-06368-s001.pdf]

Supplementary Materials

Content

1. **Scheme S1** ··S1
2. **Figure S1** ··S1
3. **Table S1** ··S2
4. **Copies of ^{13}C-NMR spectra of all compounds** ·························S3–S4

Scheme S1. Synthesis of 18*β*-GAMG, 18*α*-GAMG and 18*α*-GAMG.

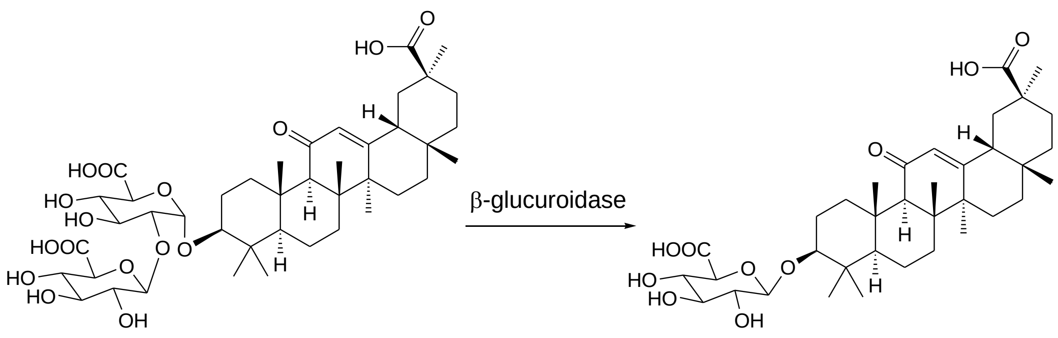

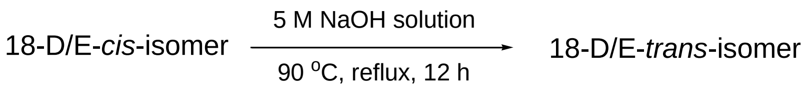

Figure S1. The isomerization reaction was monitored by ^{13}C-NMR spectroscopy.

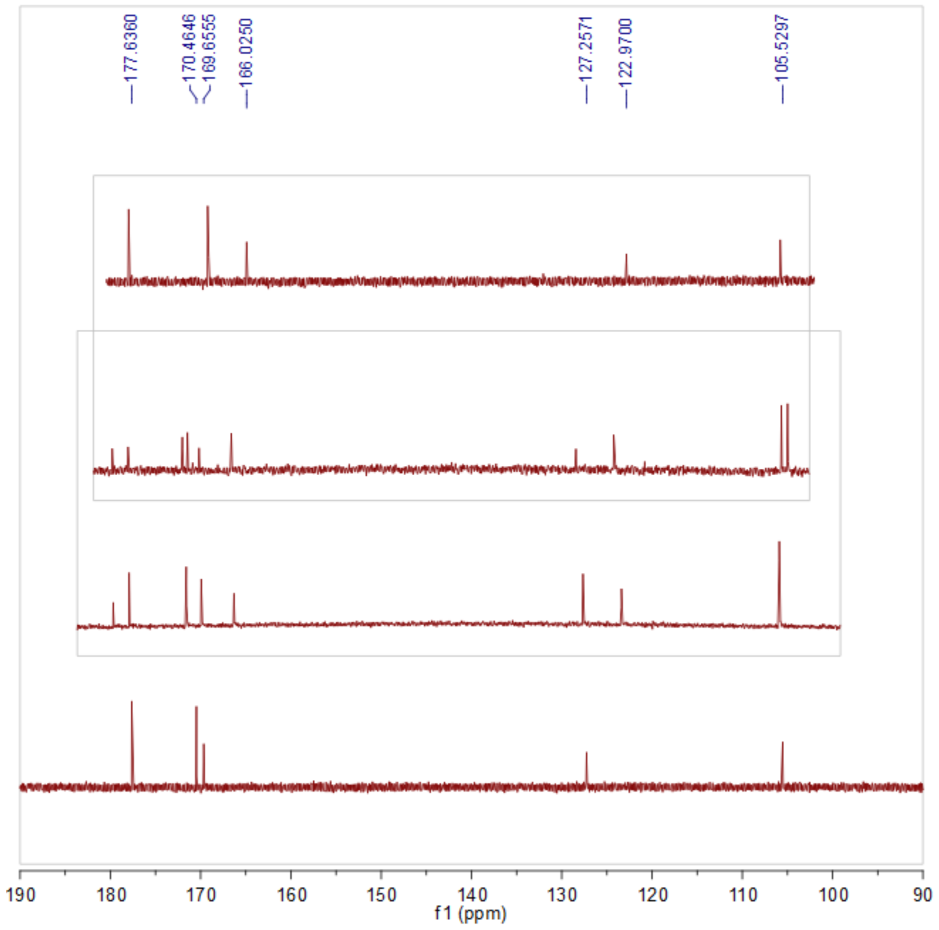

Table S1. ^{13}C-NMR data for 18*α*-GA, 18*α*-GAMA, 18*β*-GA and 18*β*-GAMG in DMSO-d_6 (*δ*, ppm, *J* in Hz).

Position	18*α*-GA	18*α*-GAMG	18*β*-GA	18*β*-GAMG
1	38.6	38.3	38.7	38.4
2	25.5	25.7	25.8	25.6
3	88.3	87.9	88.3	87.7
4	39.0	39.1	39.1 [a]	39.1 [a]
5	54.4	54.3	54.4	54.1
6	17.1	17.1	17.0	16.9
7	33.2	33.2	32.2	32.1
8	43.4	43.4	44.9	44.9
9	59.9	59.9	61.1	61.0
10	36.2	36.2	36.4	36.3
11	198.8	198.7	199.1	198.9
12 [*]	123.1	123.1	127.4	127.3
13 [*]	166.2	166.1	169.8	169.7
14	44.7	44.7	43.0	42.9
15	26.3	26.3	26.1	26.1
16 [*]	36.8	36.7	25.9	25.8
17 [*]	35.4	35.3	31.6	31.5
18 [*]	39.6 [a]	39.8 [a]	48.1	48.0
19 [*]	31.5	31.4	40.7	40.6
20 [*]	41.7	41.6	43.2	43.1
21 [*]	28.5	28.4	30.4	30.4
22	35.2	35.1	37.6	37.5
23	27.3	27.5	27.2	27.4
24	16.2	16.45	16.0	16.2
25	16.4	16.49	16.3	16.4
26	18.2	18.2	18.4	18.3
27 [*]	20.4	20.4	23.0	23.0
28 [*]	15.7	15.7	28.5	28.4
29 [*]	20.7	20.6	27.9	27.8
30	179.7	179.5	177.8	177.6
1'	103.5	105.6	104.8	105.5
2'	81.0	75.6	82.7	76.1
3'	74.2	73.7	76.3	75.6
4'	72.0 or 71.9	71.6	71.6 or 71.3	71.5
5'	76.6	76.1	74.9	73.7
6'	172.4	170.6	172.4	170.5
1''	103.7		103.5	
2''	75.0		75.7	
3''	75.2		75.9	
4''	72.0 or 71.9		71.6 or 71.3	
5''	76.0		75.2	
6''	172.0		172.0	

[*] Carbon atoms of chemical shifts with significant difference; [a] Overlapped with solvent.

Copies of ^{13}C-NMR spectra of all compounds

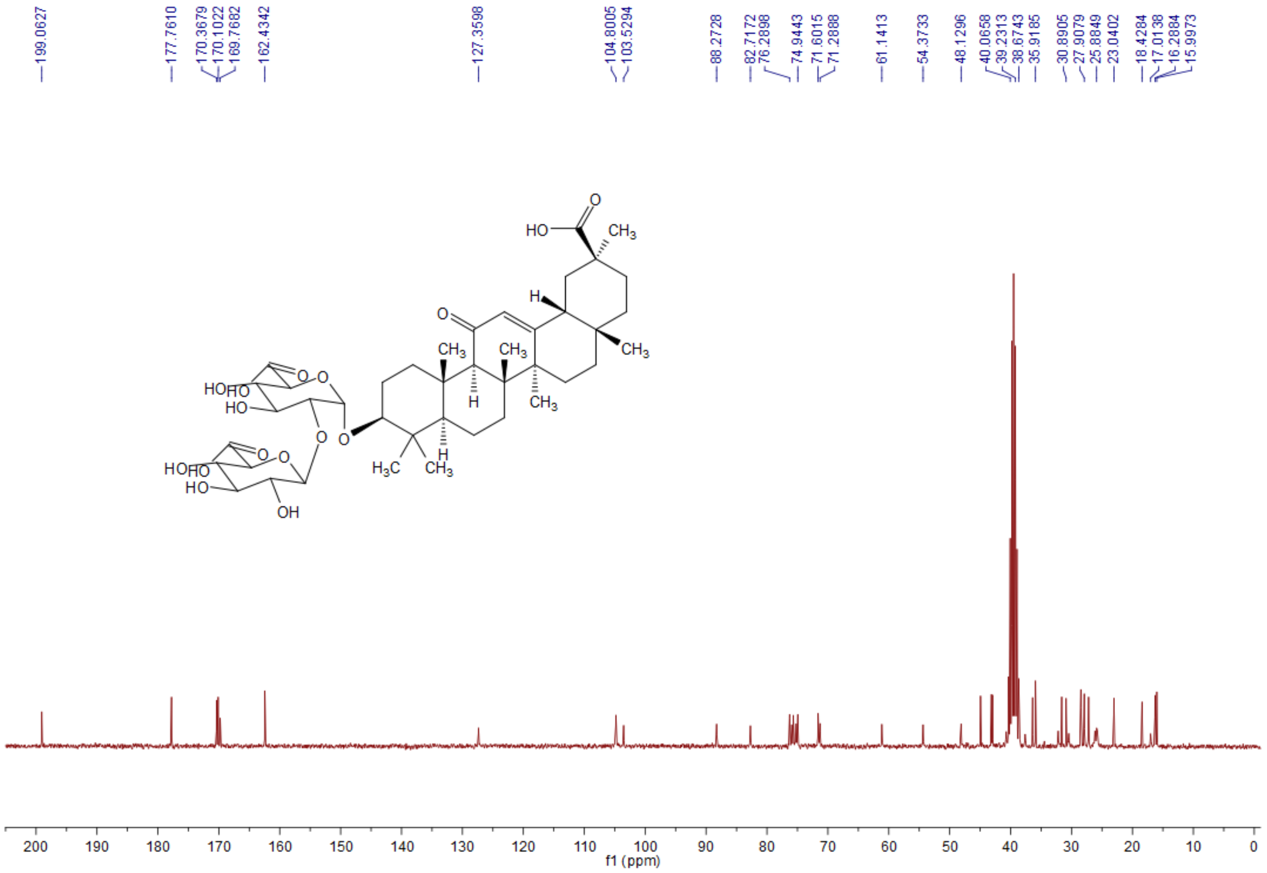

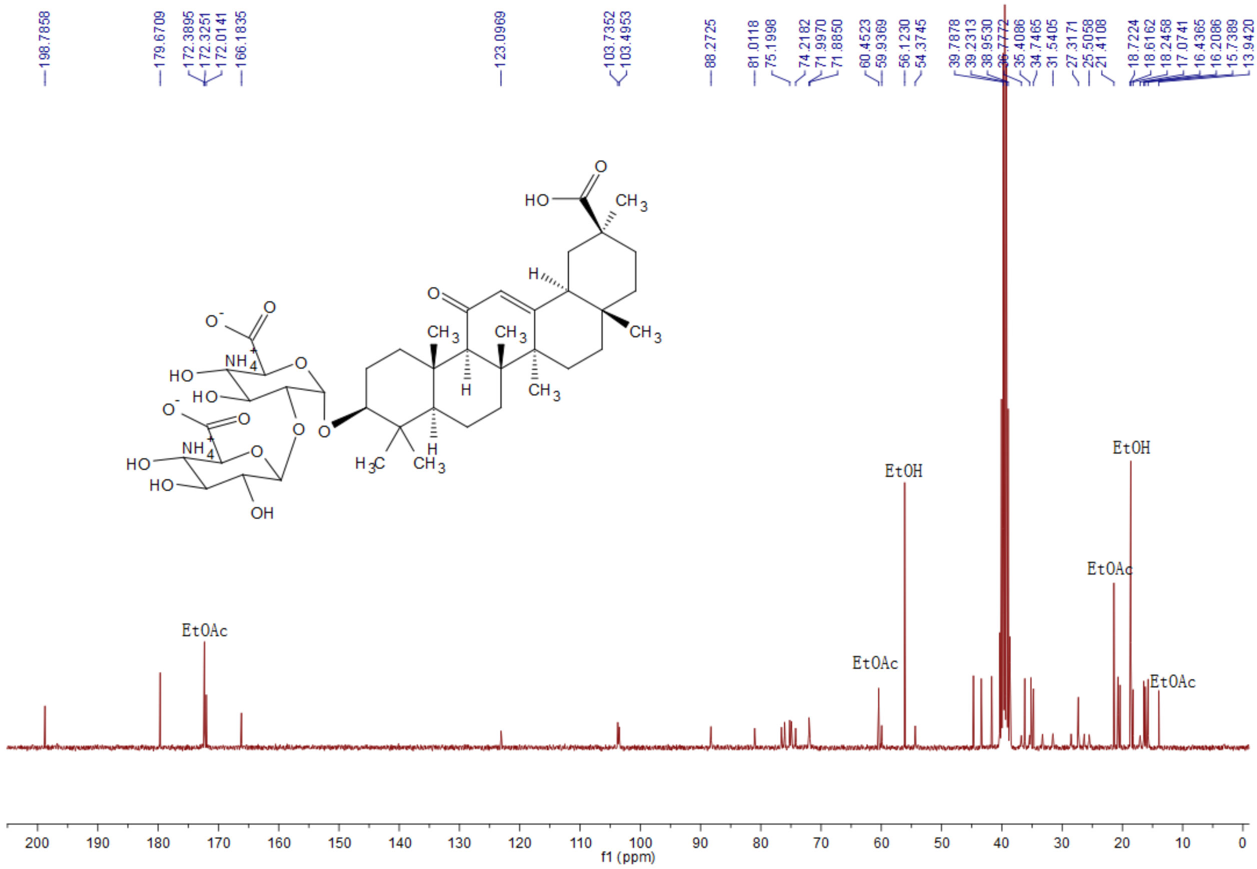

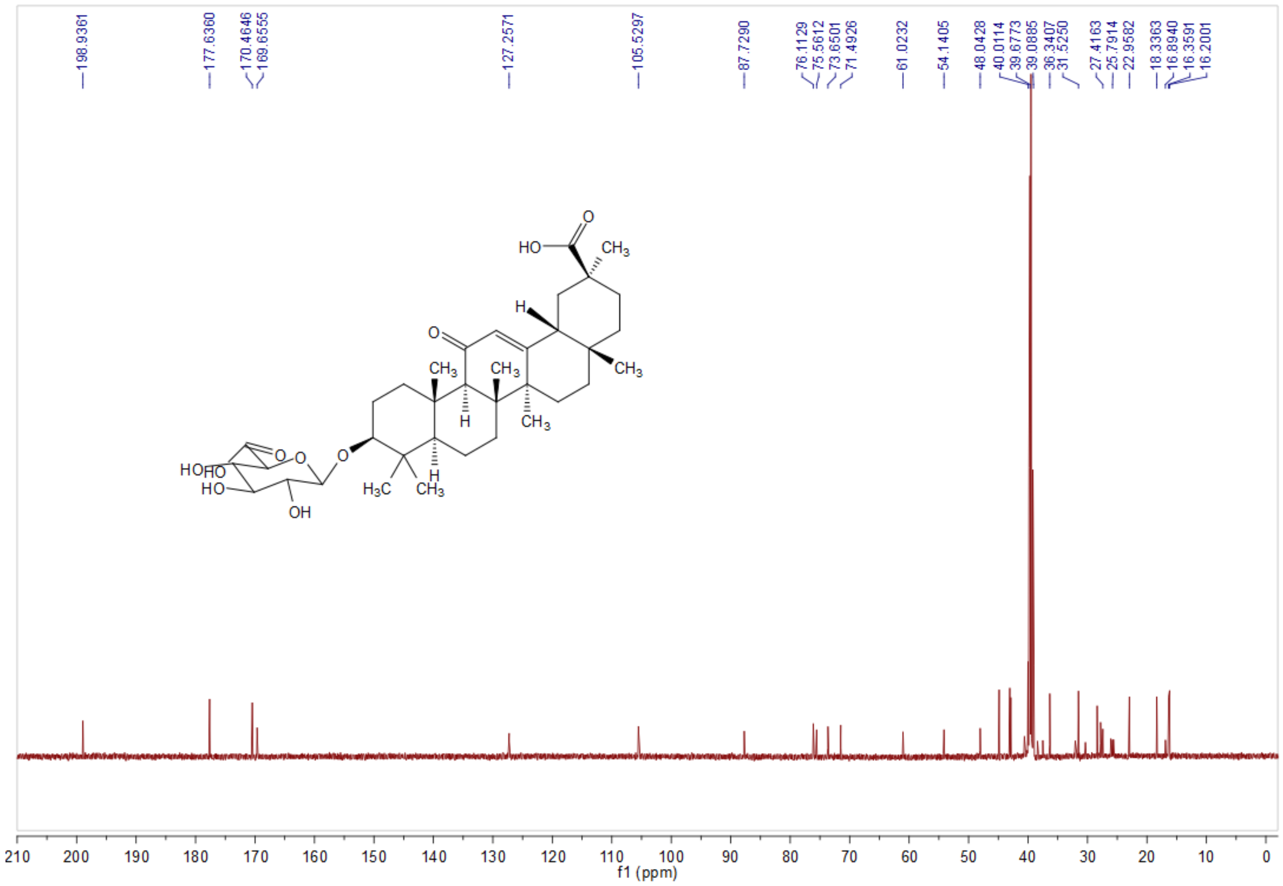
198.9361
177.6360
170.4646
169.6555
127.2571
105.5297
87.7290
76.1129
75.5612
73.6501
71.4926
61.0232
54.1405
48.0428
40.0114
39.6773
39.0885
36.3407
31.5250
27.4163
25.7914
22.9582
18.3363
16.8940
16.3591
16.2001
f1 (ppm)

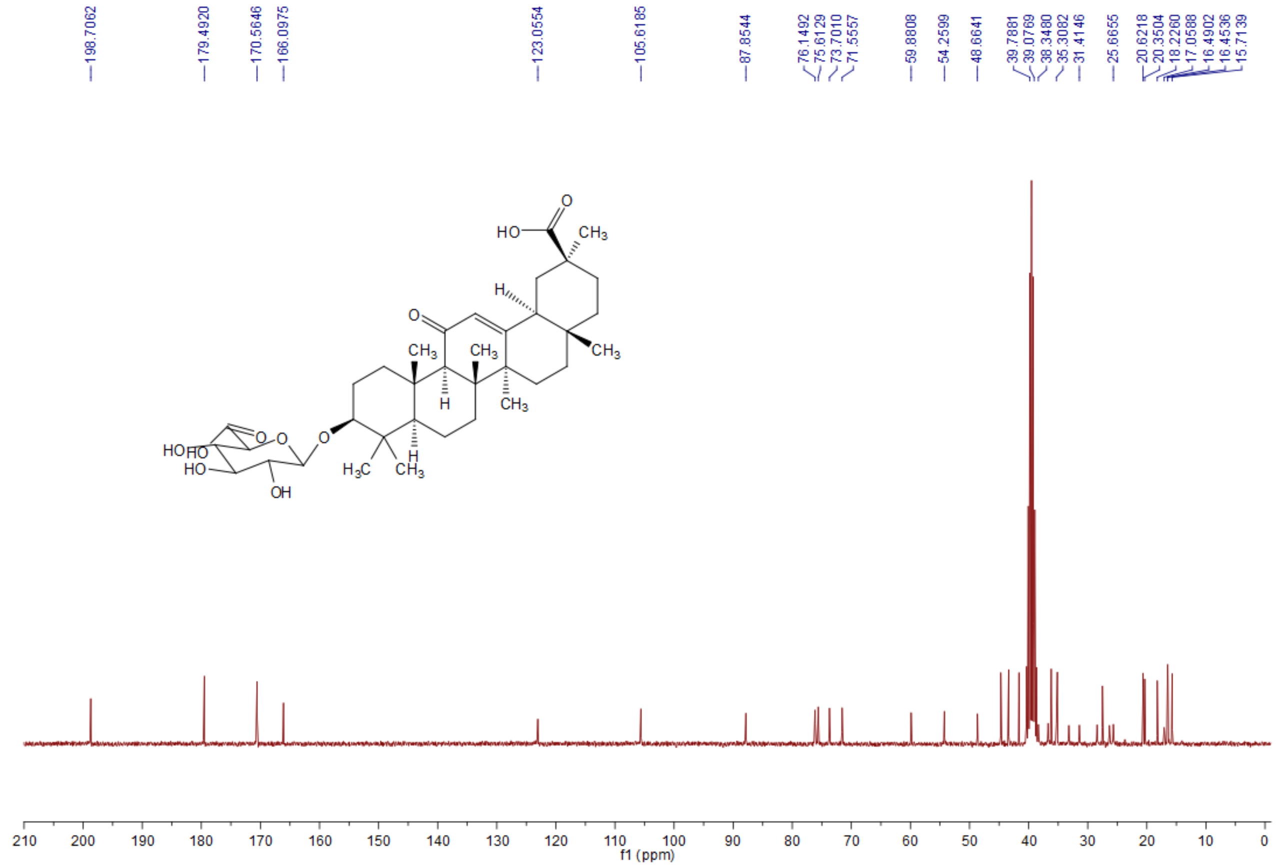
198.7062
179.4920
170.5646
166.0975
123.0554
105.6185
87.8544
76.1492
75.6129
73.7010
71.5557
59.8808
54.2599
48.6641
39.7881
39.0769
38.3480
35.3082
31.4146
25.6655
20.6218
20.3504
18.2260
17.0588
16.4902
16.4536
15.7139
f1 (ppm)
